# A Refrigerated Web Camera for Photogrammetric Video Measurement inside Biomass Boilers and Combustion Analysis

**DOI:** 10.3390/s110201246

**Published:** 2011-01-25

**Authors:** Jacobo Porteiro, Belén Riveiro, Enrique Granada, Julia Armesto, Pablo Eguía, Joaquín Collazo

**Affiliations:** 1 ETS Ingenieros Industriales, University of Vigo, Lagoas-Marcosende s/n 36200—Vigo, Spain; E-Mails: porteiro@uvigo.es (J.P.); egranada@uvigo.es (E.G.); peguia@uvigo.es (P.E.); joaquincollazo@uvigo.es (J.C.); 2 ETS Ingeniería de Minas, University of Vigo, Lagoas-Marcosende s/n 36200—Vigo, Spain; E-Mail: belenriveiro@uvigo.es (B.R.)

**Keywords:** biomass boiler, web camera, photogrammetric processing, CFD analysis

## Abstract

This paper describes a prototype instrumentation system for photogrammetric measuring of bed and ash layers, as well as for flying particle detection and pursuit using a single device (CCD) web camera. The system was designed to obtain images of the combustion process in the interior of a domestic boiler. It includes a cooling system, needed because of the high temperatures in the combustion chamber of the boiler. The cooling system was designed using CFD simulations to ensure effectiveness. This method allows more complete and real-time monitoring of the combustion process taking place inside a boiler. The information gained from this system may facilitate the optimisation of boiler processes.

## 1. Introduction

As the third primary energy resource in the world, biomass currently accounts for approximately 14% of the world’s energy consumption; a share higher than coal (12%) and comparable to gas (15%) [1]. Combustion of biomass in power plants seems to be a promising technique, both to overcome the greenhouse effect and to act as a solution for waste disposal [2]. Renewable fuels, such as biomass, have chemical-physical properties that vary greatly and may cause a process behaviour that is difficult to handle and causes fluctuations in the location and time of the drying, ignition and burning. Heat release, temperatures and off-gas composition fluctuate, thereby having a negative effect on burnout, off-gas composition and energy efficiency [3]. Improvement of biomass fuel combustion necessitates continuous process monitoring [4].

Optimum biomass combustion requires quick adjustment of the control system to handle fluctuations and adapt to them in near-real time. There is evidence that shows the geometric, luminous and fluid dynamic characteristics of flames in combustion systems are directly linked to combustion efficiency, pollutant emissions and furnace safety. Advanced monitoring and characterisation of such flames are vital for understanding and optimising combustion processes [5].

Conventional measurements are monitored by pyrometers, thermocouples and off-gas probes, but fuel-induced fluctuations cannot be detected in real-time with a highly sensitive resolution with these techniques. These techniques make measurements at specific points so they are not considered to be representative of the whole process. If several thermocouples were used, the temperature profile of the combustion gas along the refractory walls could be obtained; however, the technique would not be viable to determine the temperature profile during combustion throughout the solid waste bed, which would be continuously changing.

It is important to have a detailed, comprehensive temperature map, as opposed to conventional measuring devices that rely on only one spot value. A spot value is often greatly affected by errors because after several days the sensor becomes covered by a layer of ash materials, and this has an influence on the measured parameter, increasing the response time [6]. With proper calibration, these devices could provide maps of the temperature fields in combustion systems, such as boilers, or in other systems in which the evolution of temperature is an important parameter.

Recently, a great deal of research has been focused on the development of advanced instrumentation systems to be used for quantitative monitoring and characterisation of flames, especially through optical sensors, digital imaging and image processing techniques [5]. Alternatively, cameras are quickly able to detect the combustion state with the necessary high resolution without requiring any contact or causing any interference. Using cameras, changes can be detected at an early stage and this information can be used for monitoring or for specific combustion control actions. Two-colour radiation and pyrometry devices have previously been developed for continuous measurement of 3D temperature distributions in combustion flames [5]. Other applications with infrared and video cameras are presented in [3,4]. FTIR (Fourier transform infrared spectroscopy) applications to gas concentration detection using mid-infrared optical fibres have been studied in [7]. The use of a thermographic camera to produce a temperature map of a combustion chamber is presented in [6]. Temperature distribution and soot concentration of flames measured by the two-colour principle with an endoscope, an optical assembly with optical filters and a CCD camera is presented in [8]. Huang *et al.* [9] present two-dimensional temperature measurements of open flames by dual-spectral image analysis. Beheemul *et al.* [10], Gilabert *et al.* [11] and Lu *et al.* [12] presented a methodology for three-dimensional (3D) visualisation and luminosity reconstruction of combustion flames. Their method uses three monochromatic images acquired from three different synchronised cameras. A 3D model of the flame is reconstructed based on the 3D modelling principles of photogrammetry. Then physical properties, obtained by means of image processing, are incorporated into the 3D model of the flame.

Martinez de Dios *et al.* [13] showed a methodology for fuel bed 3D reconstruction by means of image acquisition with two cameras. A stereoscopic photogrammetric method for temporal monitoring of flames in open combustion processes is presented in [14] and [15]. In this case, the 3D position of points in the flame contours are obtained after the application of an algorithm based on cross correlation matching of images. The application of photogrammetric procedures to the rectification of combustion elements images can also be seen in Pastor *et al.* [16].

Optical devices always have a fixed position, which hinders the observation of different sights with the same camera. Furthermore, these devices were developed for high power systems or for research purposes; they are expensive and difficult to implement in low power biomass boilers (less than 100 kW).

This paper describes a prototype instrumentation system for geometric ash layer and bed measurements, as well as flying particle detection and pursuit using a single device (CCD) web camera. By means of a photogrammetric procedure based on 3D image resection some of these parameters were geometrically measured. The possibility of moving the camera to different view angles increases the monitoring potential. The low cost of the instrumentation and the robust construction make it easy to implement in a low power boiler for combustion diagnosis.

## 2. Experimental

### 2.1. Boiler Description

**Figure 1.**
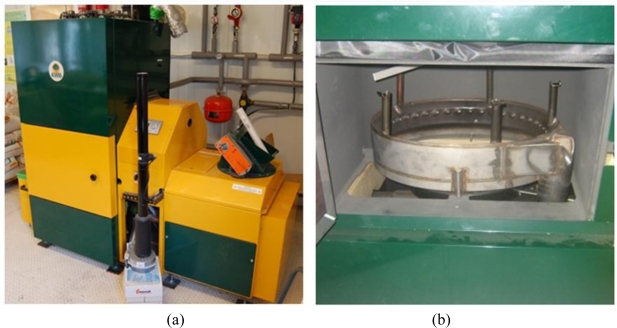
**(a)** General view of the boiler **(b)** Detail of the combustion chamber.

The low power biomass boiler plant, where the web camera was installed, is a 60 kW KWB USV–60 ZI (Figure 1).

It has an underfed feeding system with an after-burn ring. In the primary combustion area (burner plate), the fuel is fed in from underneath together with the slow preheated primary-air flow, providing gasification conditions. Due to the special arrangement of secondary air jets in the afterburning ring, a vertical cyclone with high turbulence and high combustion temperatures is reached. A high-temperature bounce dome that supplies tertiary air and increases the residence time for burnout purposes is placed on the top of the ring. This boiler was primarily designed for pellets but other biomass fuels of similar sizes can be burnt too.

**Figure 2.**
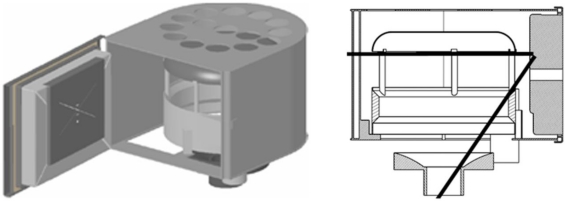
Instrumentation device.

The instrumentation device with the web camera is placed in the front access door of the boiler, just in front of the burner (Figure 2). This is an appropriate position for capturing the largest section of the combustion chamber, which facilitates a fair 3D visualisation. Consequently, the web camera is placed on a vertical wall, with the possibility of varying its vertical position and the viewing angle. This camera position was selected in order to minimise irreversible boiler alterations.

### 2.2. Instrumentation Device: Web Camera Housing and Mechanism

**Figure 3.**
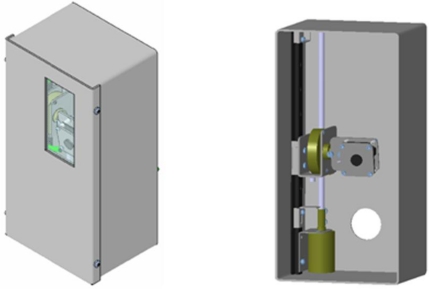
Web camera housing consisting of a guide rail, threaded rod, limit switch, lift support, electrical engines and web camera.

A schematic representation of the instrumentation device is given in Figure 3. The vision system will be located in a cockpit that will be sealed for corrosion-avoidance and refrigeration reasons. The cockpit replaces an insulator component and requires only minor modifications to the front boiler door. The cockpit has a hole in its back side, which aligns with the viewfinder with the door. The glass cover makes camera vision possible. The hole located in the back of the cockpit is where the electrical cables and refrigeration hose pass, allowing external communication. The cockpit is made from 2 mm thick stainless steel plate and supports the other pieces of the system. In order for the camera to see, the front of the system must be protected. Inactinic glass is used because the environmental temperatures are near 800 °C in air and 1,000 °C with flame radiation. These temperatures must not affect the transparency of the cover in order to ensure image integrity. A weld crystal was chosen that, due to its tonality, Protects the interior of the cockpit from part of the radiation. This weld crystal is as well protected from potential physical damage, as are the other transparent non-actinic crystals.

The system consists of a compact video camera with ample view angles and two small electrical motors with reduction that allow high accuracy in positioning the camera; one changes the vertical direction and the other varies the angle of sight. These elements require other mechanical elements for proper connection.

The stepper motor that turns the camera performs movements at 1.8° amplitude intervals. It is able to fix a position due to its torque of retention. By analysing the maximum torque that could be exerted by the camera on the axis of the motor, we determined that 0.02 Nm retention of the motor was sufficient.

### 2.3. Instrumentation Device: Web Camera

The web camera, model GANZ CMH212, is a commercial high resolution colour camera with a sensor resolution of 0.44 million pixel that was connected to a personal computer, which saved and processed the colour images taken by the 1/4” IT CCD (Charged Coupled Device) sensor. A commercial capture video card was connected to the personal computer and, consequently, a continuous digital video could be displayed in real-time on the remote personal computer with a maximum frame rate of 30 fps.

### 2.4. Instrumentation Device: Cooling System

The cooling system consists of two parts, the temperature controller and the cooling devices. The temperature controller is comprised by a K-type thermocouple coupled to the integrated circuit of the camera. The integrated circuit is the most delicate part of the camera. Therefore, if the temperature rises above 70 °C, the camera automatically descends out of the glazed zone, thereby avoiding the radiation.

**Figure 4.**
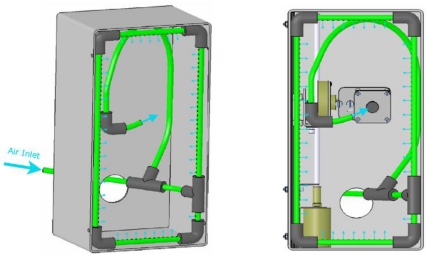
Cooling system.

Refrigeration is realised by means of compressed air provided at a constant pressure of 2 bar. The refrigerant enters the cockpit through a 6 mm pipe that is divided into two branches. The first branch is formed by a flexible tube so it can follow the camera movements. This branch cools the integrated circuit of the camera. The second branch creates an air film at the face of the crystal, which is the hottest zone due to the radiation of the flame. This film is obtained by means of four pipes perforated along their length. Details of the cooling system are shown in Figure 4.

The cooling system is a critical part of the instrument. Thus, a CFD (Computational Fluid Dynamics) analysis was made to predict cockpit temperature distributions. Fluent 6.3 software [17] was used in this simulation; k-ɛ standard and Discrete Ordinates were the turbulence and radiation models, respectively [18]. CFD analysis of the camera system and its mechanisms was developed by simulating the conditions inside when the door of the boiler was in place. The entire assembly is thermally insulated so it can be considered to be adiabatic except at the glass where heat transfer must be considered. Gases around the box have temperatures in the vicinity of 900 K and the radiation equivalent flame temperature is about 1,400 K [19,20]. These data were extracted from a simulation of the whole boiler operating under nominal conditions [21,22].

### 2.5. Instrumentation Device: The Photogrammetric System

**Figure 5.**
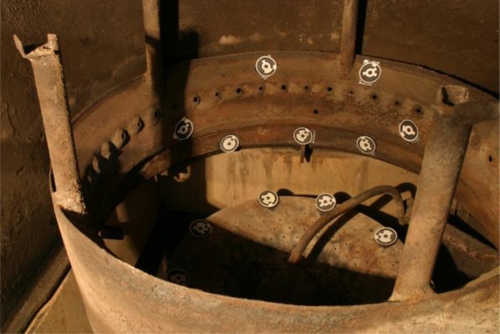
Boiler with coded targets.

To geometrically model the combustion process, a system to make metric measurements was designed. This system was based on convergent photogrammetry principles (Luhmann *et al.* [23]). Combustion modelling inside the boiler was carried out in two steps. First, a photogrammetric survey with conventional photogrammetric cameras was done to create a 3D·model of the combustion chamber. Second, the combustion process was monitored using a web camera, which was registered with the model by using checkpoints within the boiler (Figure 5).

#### 2.5.1. Modelling the Boiler Interior with a Conventional Camera (Canon EOS 10D)

In the first phase we used a semi-metric camera (Canon EOS 10D) with a 6.23 megapixel CCD sensor and RGB matrix. The lens used is a Canon Ultrasonic EF 20 mm f/2.8. To get the orientation of marked points, coded targets in the RAD model with 32 mm outside diameters were used. These targets were created with Photomodeler Pro software. Simultaneously, fixed metallic targets were placed so that they could be identified by the web camera during the combustion processes.

The data collection method was designed based on the principles of convergent photogrammetry. Due to its complexity, the post-combustion ring was removed from the interior of the modelled boiler. The combustion chamber model was separated from the post-combustion ring. Then the ring was placed back inside the boiler and new images were formed to build a main model, which joined the two previous models. Once the pictures of the boiler had been taken, it was necessary to calibrate the camera for the shooting conditions. This helped determine the internal orientation of the models. Values obtained for these conditions are shown in Table 1.

The images were processed with the restitution platform Photomodeler Scanner Pro^®^. Based on the collected interior orientation data, the external orientation of each of the three models (furnace, ring and both combined) was calculated. The coded targets were used to obtain a model of the furnace and the ring together. Once all the geometric models were registered and well-oriented, a dense cloud of points to represent the combustion dome was generated automatically with the Dense Surface Modeling (DSM) software module of the Photomodeler Scanner.

#### 2.5.2. Photogrammetric Scheme of the Combustion Process

To model the combustion process and identify the different areas, a GANZ CMH212 web camera was used. The pictures used for modelling the combustion process were extracted from videos recorded during normal operation of the boiler. The images were extracted at 2 second intervals. The web camera was calibrated before its use, and the calibration parameters for the internal orientation of the web camera are listed in Table 2. To record the images of combustion with the combustion model of the plate, a spatial resection was made [23,24] by identifying the metal targets positioned in the boiler. Using this approach, each frame was externally oriented within the 3D model of the plate. Then, using a texturing plate model from the images captured by the webcam, it was possible to generate 2D orthophotos. Finally, from these orthophotos, we obtained the metrics of the combustion process at each time step [25,26].

The calibration of the camera was accomplished in a calibration field in laboratory conditions to establish focal length, principal point position and radial and tangential distortions. The exterior orientation of the camera, once installed in the boiler, was done considering the 3D coordinates of geometric singularities in its interior walls. Finally, projection planes were defined to obtain sequential metric orthophotos of the combustion process. The precision of the measurements is based on the photogrammetric calibration of the camera, not on the camera position.

## 3. Results and Discussion

### 3.1. CFD Simulation of Web Camera Housing

**Figure 6.**
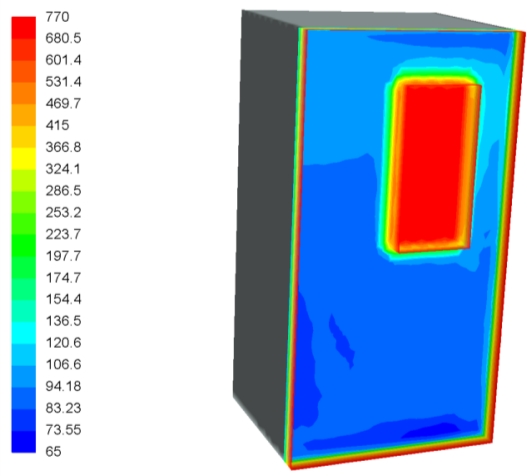
Temperature profile on the box surface (°C).

The temperature profile on the surface of the camera box is shown in Figure 6. The red rectangle in the middle of the wall corresponds to the zone where the glass is placed. Peak temperatures of 770 °C are reached in this area because of the close vicinity to the flame. The rest of the surface is much cooler than the glass because it is protected by the insulation.

**Figure 7.**
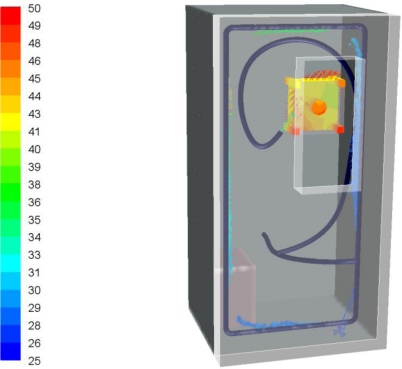
Temperature profile inside the camera box (°C).

Temperature maps inside the camera box are shown in Figure 7. There is no point in the box where the temperature exceeds the 60 °C limit imposed by the camera manufacturer. Peak temperatures in the range of 50 °C are reached in front of the camera because of the radiation emitted by the flame during combustion. Cold air leaving the refrigerating pipes is also shown in Figure 7.

**Figure 8.**
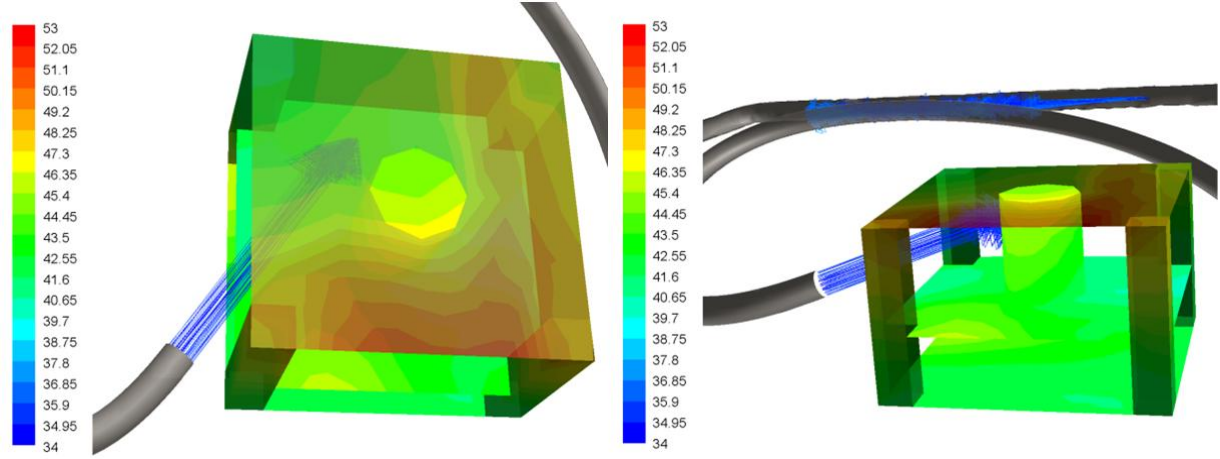
Temperature maps of the camera (°C).

Two detailed views of the camera are shown in Figure 8. This is the most sensitive part of the assembly. The temperature distribution in the camera is caused by the air flow orientation. The highest temperature in the vicinity of the camera is 50 °C.

**Figure 9.**
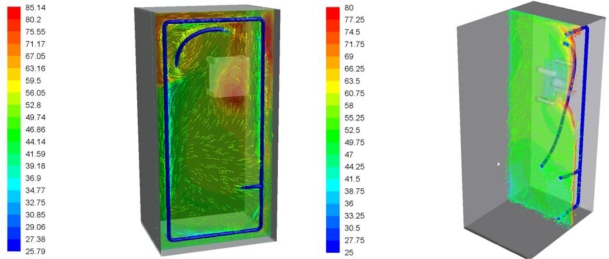
Temperature of the air flux inside the box (°C).

Figure 9 shows temperatures of the air flux inside the box. The temperature field in the plane that is coincident with the cooling air flux is represented in the detail in the left panel of Figure 9. Maximum temperatures around 85 °C are reached in this plane, especially in the regions near the glass where the cooling system has less of an effect because the cooling air flux is oriented to the camera body. The detail view in the right panel of Figure 9 represents the predicted temperature field in the plane perpendicular to the previous view.

Based on this CFD study we concluded that the assembly, with its cooling system and glass cover, would withstand the temperatures produced by the flame inside the boiler without any apparent risk of damage to the components.

### 3.2. Experimental Results

A preliminary test without the web camera installed and with a thermocouple inside the box, demonstrated that the peak temperature inside the box did not exceed 60 °C. A second test, with the web camera installed, demonstrated that the system could achieve its main objective, which was to record the pellet combustion in the bed of the boiler. We determined that the box was well located because images of the bed and both primary and secondary air injections were obtained. The cooling system did not allow temperatures to exceed 60 °C inside the box when the box was placed in the door of the boiler during the combustion process.

**Figure 10.**
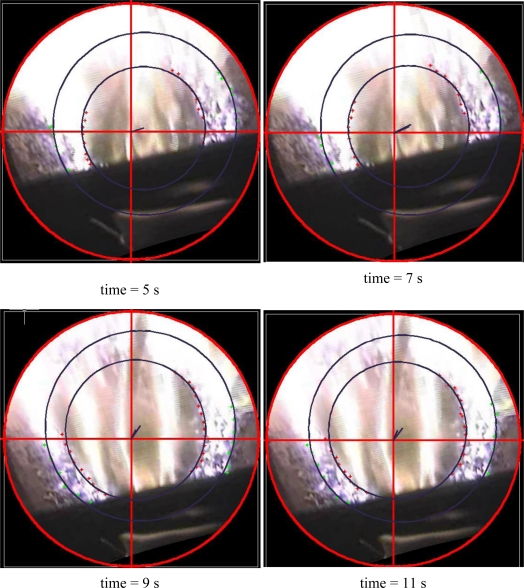

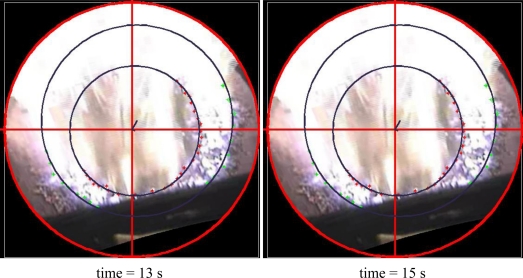
Orthophotos with adjusts at different times.

Some photos were extracted from the recordings in different stages of combustion. These photos were processed with the photogrammetric software to obtain a 3D textured model. Orthophotos, which were used to measure points and to delimit different zones, such as the combustion ring, interior and exterior, were generated from the 3D model. Once the points were measured, they were adjusted to circular forms in Matlab, so that the position of the circle centre is estimated, the radius for the inner contour of the combustion, and the centre and radius for the outer contour. These adjustments were made at 5 s, 7 s, 9 s, 11 s, 13 s and 15 s. The centre and radius adjustments can be seen in Table 3. Three different areas can be identified in Figure 10: a bright ring of char, a darker ring of char and a central homogeneous ring where drying and pyrolysis processes take place. There are some geometric parameters of the bed, such as the width of the ring of char or its displacement due to feeding, which can be observed and measured using this method. If overflow of the bed occurs it will also be detected. Ash formation and its melting and sintering in the bed during the combustion process can be studied and quantified with this method.

The combustion ring reduces its thickness as time advances, this behaviour continues until more fuel is fed in the bed. At this time, fresh pellet is introduced in the core of the bed displacing the pellet in different stages of degradation. Fuel in last phase of pyrolysis is forced to enter in the ring of char oxidation, increasing the ring size considerably, as shown in Table 3.

To estimate the symmetry of the ring on the plate, with respect to its centre, displacement vectors of the ring were calculated by taking the real centre of the plate as the origin and the estimated centre as the end point, both for the interior contour and the exterior contour of the ring. Displacement vectors for all time intervals are shown in Table 4.

At a 5 s point, the magnitude of the displacement vectors (*i.e.*, asymmetry) is greater than at 15 s. This may occur because, when the char is running out and the ring reduces its thickness, an asymmetric behaviour occurs in the combustion of the ring (the centre of the ring remains away from the central point of the dish). As the char is consumed, the depth of the bed is reduced and the non-symmetric behaviour of the bed boiler is accentuated because the air flow through bed areas which were slightly favoured is increased causing a more intense flame that strengths the consumption of fuel in these zones. This behaviour changes when fuel re-enters and combustion behaviour of the bed is re-equilibrated (the thickness of the ring increases and the displacement vector of the ring decreases).

The outlines of flames can also be seen. However, there are no flying particles greater than 1 mm and the fuel is distributed evenly around the perimeter of the burner. Images can also be used to account for the time spent by a particle of biomass in its combustion and to note that there are some particles of un-burnt pellet that pass through the burner.

Dynamic behavior of the bed has been analyzed and results of the primary tests have been exposed. The movement of the active combustion zone in the bed enlarges as the feeding system introduces pellet in the bed and the relative movements of the bed is visualized and studied thanks to the measurement system installed.

## 4. Conclusions

The paper introduced a low cost remote device for real-time monitoring of the combustion process in boilers using a web camera. The camera was mounted on the door of the boiler to gain an appropriate perspective of the combustion chamber. CFD simulations of the system under working conditions were developed to design the cooling system. These simulations demonstrated that the temperature requirements of the camera would be achieved.

The absence of time delays and the non-intrusive approach of the technique are distinctive and promising features. The photogrammetric processing of web camera images is shown to be a feasible technique for measuring combustion processes, identifying conversion zones (drying, devolatilisation and char oxidation), evaluating asymmetries in time and detecting and monitoring outgoing particles. Moreover, the real-time processing of the resulting algorithm is interesting for control purposes. As a matter of fact, once the surface and gas temperatures are known, it is possible to use that information to develop a reliable and useful control strategy. From this information, it is possible to identify hotspots or zones where the waste is drying, gasifying, igniting, burning or cooling. Consequently, the movement of waste and flow-rates of air can be regulated.

Processed photos of the bed serve to define the different zones of combustion, their dynamic behaviour (rings of char, drying...) and their displacement due to feeding, consumption and fluid dynamic forces, which can be observed and measured using this method. Ash formation, its melting and its sintering in the bed during the combustion process can be studied and quantified with this method and also how the symmetry of the bed evolves.

Works combining different measurement techniques are ongoing. Temperatures in the burner can be measured with infrared cameras and some species can be detected by employing specific filters. Automated approaches for photogrammetric measurement of control parameters are being implemented as well.

## Figures and Tables

**Table 1.** Values of initial calibration of using conditions of Canon EOS 10D.

**Parameter**	**Value**	**Standard Deviation**

Focal distance [mm]	20.704	0.002
Principal point position (x) [mm]	11.161	0.002
Principal point position (y) [mm]	7.416	0.002
Format width [mm]	22.664	5.3 × 10^−4^
Format height [mm]	15.113	-
K1 [ - ]	2.018 × 10^−4^	1.1 × 10^−6^
K2 [ - ]	−3.532 × 10^−7^	7.5 × 10^−9^
P1 [ - ]	3.287 × 10^−5^	1.1 × 10^−6^
P2 [ - ]	−1.670 × 10^−5^	1.6 × 10^−6^

**Table 2.** Calibration Values employed during the recoding of the GANZ CMH212.

**Parameter**	**Value**	**Standard Deviation**

Focal distance [mm]	4.747	0.003
Principal point position (x) [mm]	3.223	0.003
Principal point position (y) [mm]	2.244	0.003
Format width [mm]	6.055	4.6 × 10^−4^
Format height [mm]	4.500	-
K1 [ - ]	1.363 × 10^−2^	1.7 × 10^−4^
K2 [ - ]	6.149 × 10^−4^	1.0 × 10^−5^
P1 [ - ]	7.897 × 10^−4^	3.4 × 10^−5^
P2 [ - ]	−4.258 × 10^−5^	3.0 × 10^−6^

**Table 3.** Centre and radius adjustments [mm].

**t**	**X_i_**	**Y_i_**	**R_i_**	**Residue**	**X_e_**	**Y_e_**	**R_e_**	**Residue**	**Ring thickness**

5 s	218.23	245.47	82.31	2.89	219.99	251.52	122.10	1.99	39.78
7 s	697.29	249.18	80.79	2.92	699.94	251.63	120.16	2.03	39.37
9 s	1204.11	253.12	94.16	2.51	1210.51	258.50	127.77	3.85	33.61
11 s	1679.35	254.39	92.97	2.74	1686.16	256.99	126.55	2.53	33.59
13 s	2188.86	239.48	87.24	2.18	2192.04	253.09	128.78	2.72	41.55
15 s	2703.25	239.78	87.96	3.15	2704.68	256.67	131.30	3.39	43.34

**Table 4.** Displacement vectors [mm].

**t**	**Inner Ring**			**Outer Ring**		

	**X**	**Y**	**Vector**	**X**	**Y**	**Vector**

5 s	16.923	4.587	17.531	18.687	10.640	21.504
7 s	19.016	8.300	20.748	21.669	10.753	24.191
9 s	5.7230	12.243	13.515	12.128	17.625	21.395
11 s	5.724	13.505	14.668	12.531	16.108	20.408
13 s	3.870	−1.402	4.116	7.045	12.219	14.104
15 s	5.090	−1.101	5.208	6.528	15.795	17.091
